# Effect of Overripening on the Physico-Chemical and Sensory Characteristics of Boneless, Salt-Reduced Iberian Dry-Cured Ham

**DOI:** 10.3390/foods13101588

**Published:** 2024-05-20

**Authors:** Noelia Hernández Correas, Adela Abellán, José María Cayuela, Cindy Bande-De León, Luis Tejada

**Affiliations:** Department of Human Nutrition and Food Technology, Universidad Católica de Murcia-UCAM, Campus de los Jerónimos, 30107 Murcia, Spain; aabellan@ucam.edu (A.A.); jmcayuela@ucam.edu (J.M.C.); cmbande@ucam.edu (C.B.-D.L.); ltejada@ucam.edu (L.T.)

**Keywords:** ham, processing technology, temperature increase, overripeness

## Abstract

The aim of this study was to evaluate the effect of extended maturation and temperature increase on the physico-chemical, biochemical, instrumental color and texture, sensory, and acceptability parameters of cured and boneless Iberian hams. Given the limited knowledge in this area, our objective was to develop a ham with enhanced proteolysis, potentially leading to increased bioactive peptide generation and superior sensory characteristics compared to salt-reduced counterparts. To achieve this, a batch of hams cured up to 38% loss at 30 °C and two batches cured up to 42% loss at 30 °C and 36 °C were evaluated. Results showed that the increase in processing time and temperature significantly enhanced (*p* < 0.05) ham proteolysis and amino acid content without adversely affecting its texture. No significant differences were observed in instrumental texture parameters or sensory attributes as evaluated by consumers. These processing conditions also increased the content of free amino acids, improving the product quality. Overall, these processing modifications resulted in hams with excellent sensory acceptability and enhanced bioactive potential despite the salt reduction.

## 1. Introduction

Iberian ham is a high-quality product that, due to its nutritional profile and sensory characteristics, is highly appreciated by consumers [1,2]. It is one of the leading products of the Spanish meat industry, and this can be generalized to any country in the Mediterranean basin since the demand for this product is increasing [3].

The production process of cured ham is carried out first by salting the ham, then post-salting, drying, and finally, a long maturation period [4,5].

The quality of the cured ham that is finally obtained will depend on how proteolysis has evolved during the production process in response to changes in the different factors that occur during production, such as temperature and relative humidity, excluding considerations regarding the quality of the meat utilized, which also constitutes a pertinent factor for consideration.

Throughout these stages, complex proteolysis and lipolysis reactions occur that contribute to developing the sensory profile that our ham will finally have [6]. It is mainly during maturation that proteins and lipids undergo intense processes of proteolysis and lipolysis, affecting the flavor and texture of the cured ham. Final flavor formation has to develop over a long maturation time at high temperatures [7,8]. These changes depend mainly on the duration of the ripening process, and this has been corroborated in different cured products [9,10,11,12]. Increased maturation time or overripening will lead to increased proteolysis and, consequently, to an increase in the amount of potentially bioactive peptides with beneficial properties for the consumer’s health [13].

Salt is the main ingredient, after meat, in the preparation of cured ham, which is involved in proteolysis, water holding capacity (WHC), and organoleptic characteristics. In addition, it decreases water activity (aw), thus contributing to the stability of the final product [5,14].

Throughout the processing stages, salting and drying procedures yield varying profiles of salt and water content, which undergo fluctuations over time. It is crucial to quantify and evaluate how these two variables vary in order to see their effects on the biochemical evolution and sensory characteristics that develop during ham processing; control of each stage is also necessary to obtain a tasty and safe product [5].

Today’s concern about sodium intake and its relationship with certain diseases [15] has led to new initiatives to reduce the consumption of salty foods. However, in products such as traditional dry sausages this is not an easy task, since salt plays an essential role in controlling the chemical and microbial processes in ham.

In a study by Zhao et al. [16], it was observed that the salting process inhibited the potential activity of the proteolytic enzymes cathepsin B and L to only 9.31% and 13.66%, respectively, of their original potential activity, thus confirming the inhibitory role of salt in proteolysis.

Similar results were described by Harkouss et al. [17], who corroborated the promoting role of temperature and the inhibitory role of salt and drying in increasing proteolytic intensity [18].

Water activity generally affects microbial growth and the action of certain enzymes involved in proteolytic reactions, which are responsible for the development of the flavor, aroma, and texture of ham [11,19,20].

To deal with the undesirable effects of salt reduction (reduction of flavor and aroma, undesirable effects on texture, and problems with stability and color formation [21]), several options have been proposed, one of which shows that prolonging the maturation phase results in better flavor and aroma properties in hams and also reinforces muscle firmness due to muscle endopeptidases and exopeptidases that generate a large amount of free amino acids and peptides [22,23,24,25]. It has also been described that a reduction in moisture by dehydration is linked to a more persistent aroma and other sensory features [10,25,26]. One of these studies also showed that the acceptability of ham decreased with increasing curing time. High pastiness and stickiness values seemed to be more correlated with decreased acceptability despite a significant increase in aroma. These textural changes were the result of excessive proteolysis [22].

Even so, knowledge in this area is very scarce, which is why in this work we pretend to evaluate the effect of the extension of maturation on ham characteristics, with the aim of developing a ham with high proteolysis (probably more significant biopeptide generation) and with better sensory characteristics than a ham reduced in salt due to an increase in maturation time (overripening).

Knowledge in this area is relatively scarce for boneless Iberian hams reduced in salt since it has never been done. Therefore, it is unknown what side effects would be produced if the temperature or maturation time were increased.

## 2. Materials and Methods

### 2.1. Preparation and Collection of Cured Iberian Ham Samples

A total of 48 Iberian hams were needed for the development of this study. Three batches of 16 Iberian hams each were processed.

Pork legs were first deboned and then salted with sea salts and nitrifying agents at a rate of 0.8 days/kg in a cold storage chamber at a temperature of 3 °C.

After salting, the pork legs were washed with water and, after this, a traditional curing process was carried out. The resting or post-salting stage began at 3 °C, gradually increasing to 6 °C. This processing phase ended when the Iberian hams reached 18% shrinkage.

Subsequently, the temperature was raised to 30 °C. The process ended when the pieces reached a weight loss of 38%. After this last processing, the first batch had finished its maturation process, but the two remaining batches of Iberian hams were taken to overripening, with up to 42% weight loss in each but in different processing conditions: The second batch was processed at a temperature of 30 °C and the third batch at 36 °C.

The percentage weight loss was determined by weighing each sample in triplicate during each processing stage. The results were expressed as percent weight loss, considering the fresh weight of each piece.

Finally, 8 cured Iberian hams were selected from each batch (48 hams). Samples were taken at the following stages: I: raw muscle; II: beginning of post-salting; III: end of post-salting; IV: drying phase (33% weight loss); V: final product (38% weight loss for lot I and 42% for lots II and III).

### 2.2. Physicochemical Analyses

The analysis of the physicochemical parameters of the samples was carried out in triplicate for each type of dry-cured Iberian ham.

Moisture content analysis were determined following the gravimetric procedure described in ISO1442 [27].

The determination of total nitrogen (TN) and non-protein nitrogen (NPN) was carried out by the Kjeldahl method [28]. For the preparation of NPN extract, the method followed by Abellán et al. [29] was used. The crude protein value was obtained by multiplying the TN value by 6.25. The proteolysis index was determined as the percentage of the ratio of non-protein nitrogen to total nitrogen [30].

For salt content analysis, the Volhard methodology of ISO1841-1 [31] was used and slightly modified. The Folch method [32] was used to measure the intramuscular fat content of Iberian hams.

The evaluation of water activity was carried out by gravimetry using the AOAC 920.153 method [33].

Free amino acid (FAA) content was determined following the method described by Abellán et al. [34].

### 2.3. Determination of Instrumental Color and Texture Profile Analysis (TPA)

Instrumental color assessment utilized colorimetry with a colorimeter (HunterLab, Colorflex) following the CIELab system. The outcomes were represented by the L*, a*, and b* coordinates, denoting luminosity, red–green index, and yellow index, respectively. Additionally, the saturation parameter (chroma) and hue angle (h*) values were obtained. The results were averaged from three measurements [35].

For instrumental texture analysis, a QTS-25 texturometer (Brookfield CNS Farnell, Borehamwood, Hertfordshire, England) equipped with a 25 kg load cell and a 10 mm-diameter probe was employed. Texture Pro v. 2.1 software facilitated data interpretation [36].

The biceps femoris muscle was dissected into 10 × 10 × 10 mm parallelepipeds, with three parallelepipeds per sample. Testing was conducted at a controlled room temperature of 20 °C.

The procedure involved subjecting the sample to two consecutive cycles at a constant speed of 30 mm/s, with 50% compression perpendicular to the muscle fibers.

Texture profile analysis (TPA) was conducted to assess the hardness, deformation concerning hardness, adhesiveness, cohesiveness, recoverable deformation, springiness, gumminess, and chewiness.

### 2.4. Sensory Analysis

For the sensory analysis of the dry-cured Iberian ham, a consumer test was performed with an untrained panel. The analysis was carried out following the method described by Muñoz-Rosique et al. [35] with slight modifications. For this analysis, a total of 200 anonymous participants attended on a voluntarily basis and without having received any type of prior information or training. The study was conducted in a single session where the participants evaluated the three batches of hams. The age range chosen was 18 to 55 years.

For the study, participants evaluated the samples using a questionnaire that included the hedonic assessment of appearance, color, odor, texture, salty taste, overall taste, and overall acceptability. Each attribute was scored by assigning a numerical value using a hedonic scale between 1 (I dislike it very much) to 5 (I like it very much).

The samples used for the consumer sensory test were complete slices of the cross-section of the piece, following the method established in the UNE-ISO standard 6658:2019 [37] and UNE-ISO 4121:2006 [38], which were coded with a random three-digit number.

The results were obtained by calculating the average score given to each product attribute evaluated by the consumer. To determine the preference between the three samples, the percentage values of choice of the consumer panel were obtained in each type of dry-cured ham studied.

### 2.5. Statistical Analysis

All analyses of our samples were performed in triplicate. Statistical analysis of our samples was performed using SPSS software package (version 21.0, IBM Corporation, Armonk, NY, USA). All results were expressed with the mean and standard error. The results shown in the tables are expressed with two decimal places.

To evaluate the effect of overripening, a one-way ANOVA was performed between batches I and II. The effect of temperature was also evaluated using a one-way ANOVA between hams from lots II and III.

When the effect of overripeness or temperature was significant (*p* < 0.05), the results were compared using Tukey’s HSD test. Differences were considered statistically significant when *p*-values were equal to or below 0.05. Relationships among the studied factors are presented using appropriate tables and figures.

## 3. Results and Discussion

### 3.1. Analysis of the Physical–Chemical Parameters of Iberian Ham during Processing

**Figure 1:**
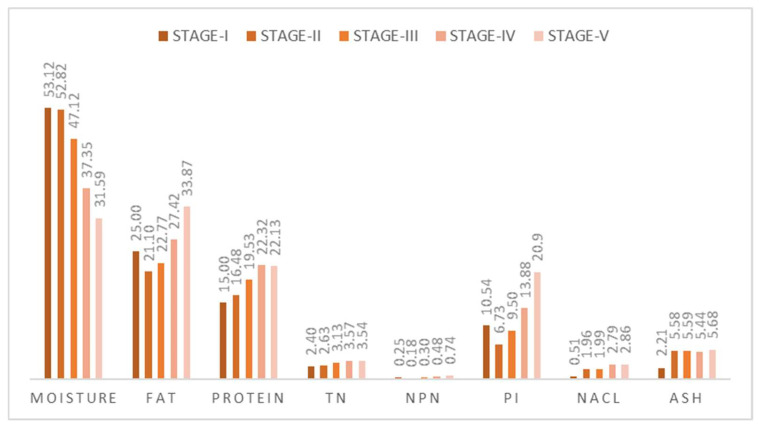
Changes in the physicochemical parameters of Iberian dry-cured ham throughout the maturation process.

Figure 1 shows the changes in the physicochemical parameters of Iberian dry-cured ham throughout the maturation process.

Figure 1 shows the evolution of the physicochemical parameters and salt content of boneless cured Iberian hams reduced in salt during the different stages of processing. The different processing stages affected all the physicochemical parameters. Moisture content in the cured ham decreased significantly (*p* < 0.001) during all stages but mainly between post-salting (stage III) and the final stage (stage V). In contrast, intramuscular fat content increased (*p* < 0.05), as did protein values. This parameter increased progressively throughout all the stages, with significant differences being observed between the raw muscle (stage I) and the final product (stage V) but not between the final stages of processing (stage IV and stage V). The same can be observed in the total nitrogen (TN) or non-protein nitrogen (NPN) content, where the evolution throughout processing was gradual, with significantly higher values (*p* < 0.05) being observed mainly between the end of post-salting (stage III) and the end of processing (stage V).

Consequently, PI increased significantly during the different processing stages (*p* < 0.05). The increase in temperature that occurs during the final stages of processing (stage IV and stage V) was directly related to the increase in temperature that occurs during these stages of ham processing, resulting in an increase in the activity of proteolytic enzymes [23,25,29].

The physicochemical modifications that took place during processing coincide with those observed during the processing of other cured hams [26,35].

NaCl concentration increased throughout processing but mainly at the beginning of the post-salting stage (stage II). This significant increase was due to the incorporation of curing salts associated with the manufacturing process (*p* < 0.05). An increase in NaCl values was observed between stages I and II, as well as between the end of post-salting and the last two stages (stages IV and V), which was due to the decrease in moisture content, which takes place during last stages of ham processing. The final salt content indicates a correct diffusion of salt, which is important not only for the texture and flavor of the ham but also to ensure the microbiological quality of the ham [14,19,35]. The ash content remained stable throughout processing, and significant differences were only observed between stage I (raw muscle) and stage II (beginning of post-salting).

### 3.2. Effect of Change in Processing Conditions in the Storage Phase on the Physicochemical Parameters of Iberian Ham

Table 1 shows the effect of the overmaturation along with the increase in the temperature on the physicochemical parameters obtained from Iberian ham.

Table 1 shows the results obtained for the different physicochemical parameters with the different processing conditions. In batch I, the final percentage of weight loss or loss was 38% at 30 °C, and for batches II and III the final percentage of weight loss was 42% in both batches, at 30 °C for batch II and at 36 °C for batch III.

Overripening had a significant effect (*p* < 0.05) on moisture content, with the lowest moisture content being obtained in the hams of lot II as a result of the increase in ripening time, which is consistent with the progressive weight loss experienced by hams with continued ripening [9,12,24].

The salt content of the hams was not significantly affected by the different processing conditions; a slight increase in salt content was observed between hams from lots I and II. A more marked increase in salt content was observed between hams from lot I and lot III. The increase in salt content was due to the increase in the maturing time of hams from lots II and III. An increase in curing time causes the water content to decrease and, consequently, the salt concentration increases [18,39]. Proteolysis in hams can be affected by humidity and salt content. A high salt content could slow down the activity of proteases, but this was not the case with our hams, which were reduced in salt [40,41].

Overripening also significantly affected the intramuscular fat content of the ham, with significantly higher values being obtained in lots II and III compared to lot I. Other studies carried out on Jinhua hams related this increase in processing time and temperature increase to the acceleration of lipid oxidation and a higher concentration of volatile components directly related to flavor development [7].

Ash content increased significantly in lots II and III with respect to lot I due to overripening, which decreases the moisture content of the ham [42].

### 3.3. Effect of Processing Conditions on the Biochemical Parameters of Boneless Iberian Ham during the Final Storage Stage

**Figure 2:**
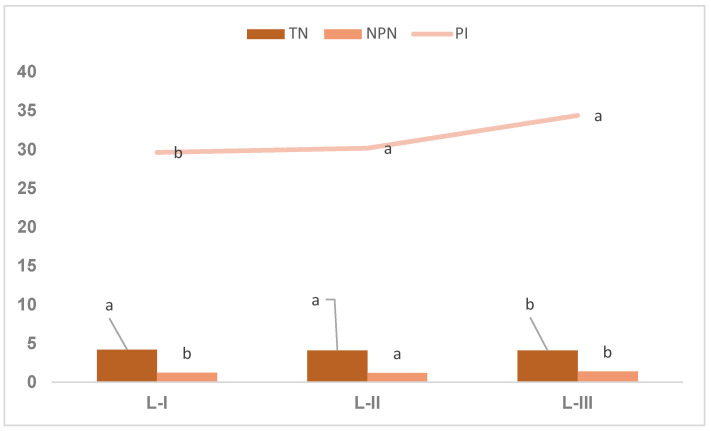
Changes in biochemical parameters with different processing conditions of Iberian dry-cured ham. Values within a row with different superscripts differ significantly at *p* < 0.05.

Figure 2 shows the effect of changing the processing conditions of Iberian ham on biochemical parameters.

Figure 2 shows the biochemical content of boneless cured Iberian hams reduced in salt in the final stages of processing of the different batches. Total nitrogen and protein content did not vary significantly despite the changes in processing time and temperature applied to batches II and III. Similar values were obtained for the three batches in both parameters studied.

The proteolysis index was significantly affected (*p* < 0.05) by the processing changes in the ham. Hams from lots I and II obtained values similar to each other and significantly lower than those of hams from lot III. This was due to the increase in temperature in the final phase of the Iberian hams from lot III, which may have resulted in the significant variations that took place during proprotein degradation, which induces protease activity [5]. Similar results were observed in other studies, where increasing the temperature and drying level significantly (*p* < 0.05) increased the NPN content and the IP [43,44,45]. The hams from batch II were not affected by the increase in processing time for the hams from batch I, and this may have been because there comes a moment in which, despite continuing with the processing, proteolysis remains stagnant and is not favored by the increase in processing time.

Non-protein nitrogen content was not significantly affected by the different processing conditions; higher values were observed in the hams of lot III, which had up to 42% weight loss or loss at 36 °C. This may have been due to the increase in the temperature of the cellar in the final phase, since increasing the temperature increased proteolysis and, with it, the activity of cathepsins B and L, which contribute to the production of smaller peptides and free amino acids, which also contribute to the development and quality of the cured ham [6]. Similar results have been obtained for Jinhua hams [46].

The non-significant results for these fractions (TN and NPN) and the similar results for all batches can be explained by the fact that we know that over-translation and temperature increase protein degradation. Thus, the salt content in all hams was reduced, which also increases cathepsin activity [16,46].

Results for the effect of different processing conditions on Iberian dry-cured ham on free amino acid (FAA) content in the final product are shown in Table 2.

Changes in the concentration of free amino acids also indicate changes in ham proteins during storage [47]. The different processing conditions of the salt-reduced boneless cured Iberian ham significantly affected the composition of free amino acids. No values were detected for the amino acids aspartate (Asp) and cysteine (Cys), coinciding with the results obtained in a study on lamb ham, where an increase in maturation time resulted in undetectable values of this amino acid [47]. Serine (Ser), methionine (Met), arginine (Arg), phenylalanine (Phe), and lysine (Lys) were not significantly affected by the different processing conditions (overripeness and temperature) to which the different batches were subjected. Similar but slightly higher values were observed in almost all the hams from batch I, except for phenylalanine and lysine, where the highest values were obtained from batches II and III. These results coincide with those found in another study, where increased storage time increased the concentration of these amino acids [47]. For the rest of the free amino acids, temperature and over-maturing led to significant changes in the free amino acid content. For histidine (His), glycine (Gly), alanine (Ala), tyrosine (Tyr), and valine (Val) overripening and temperature produced significant changes (*p* < 0.05) in the concentration of these amino acids. Glutamic acid (Glu), tryptophan (Tro), and proline (Pro) were also significantly affected (*p* < 0.05) only by overripening, without the increase in temperature, producing significant changes in their concentration. In the case of threonine (Thr), isoleucine (Ille), and leucine (Leu) it was temperature that produced significant differences (*p* < 0.05) in their concentration. For all free amino acids, these processing changes favored a significant increase in their concentration, with the hams of batch III being in most cases the ones that showed the highest concentrations. Therefore, peptidase activity was significantly affected by the increase in temperature during the final phase and overripening [4], as was the total free amino acid (FAA) content, which was significantly higher in batches II and III than in batch I, with batch III having the highest values.

The results obtained for batch I are similar to those obtained in the study by Muñoz-Rosique et al. [35] for traditionally cured hams. In the first place, the reduction in salt did not cause any decrease in the amino acid content, as corroborated by the study by Cittadini et al. [48].

FAAs are also a type of taste substance. Glutamic acid is the amino acid associated with the umami taste of Jinhua ham [7]. In addition, alanine is associated with sweet taste and leucine with bitter taste. A decrease in their concentration during ham maturation can diminish these flavors [47], something that did not occur in our hams since overripening and temperature had the opposite effect of increasing the concentrations of these amino acids. Lysine is related to the flavor of ripening [7,47], coinciding with the results obtained for our hams, where the lysine values for lots II and III were the highest.

In addition, it was observed that, with respect to the amino acid content of the hams from batch I, there was an increase in the concentration of almost all amino acids, reflecting the fact that the temperature and overripening favored the activity of the peptidases. Similar results were observed in another study, where processing time increased the total free amino acid content in Jinhua hams with increased maturation time and processing temperature [7].

A higher content of free amino acids is directly related to an increase in the substrate of the Strecker degradation reaction, which is responsible for the formation of aromatic molecules that directly contribute to the flavor of cured ham [8,49].

### 3.4. Instrumental Color and Texture Profile

Table 3 shows the effect of different processing on the instrumental color obtained from Iberian ham.

Instrumental color parameters were not affected by the temperature or overripeness of the cured Iberian ham (*p* ˃ 0.05 in all cases), coinciding with the data obtained in similar studies [22]. The brightness value (L) was slightly higher in the hams from lot I, as well as in the h parameter. A decrease in L* values was directly related to the decrease in moisture or, in other words, the dehydration of the cured ham, since a decrease in water increases the concentration of pigments such as nitrosyl-myoglobin. As a consequence, there is a reduction in luminosity (L*) [50]. The range of values for parameters a, b, and c was similar for all the batches of hams, so they were not significantly affected by overripeness or increased temperature.

Table 4 shows the effect of different processing conditions on the instrumental texture of Iberian dry-cured ham.

Overripening and the increase in processing temperature in the final phase did not have a significant effect on the different parameters defined for the instrumental texture. Only the adhesiveness was significantly higher in hams from lots II and III with respect to lot I, with lot III obtaining the highest values. Although there were no significant differences, for the rest of the parameters, slightly higher values were observed for hams from lots II and III with respect to lot I. Higher hardness values have been attributed to water content, since texture is positively related to moisture loss in ham [10]. As a general rule, the lower the moisture content, the better the consistency [51]. Despite showing greater proteolysis, the texture parameters analyzed did not indicate a softer texture. This could be due to the positive effect of temperature and overripening on hardness, since for both batches (II and III) the hardness was higher than for batch I, with these results also coinciding with those obtained in other studies [9,43].

Table 5 shows the effect of different processing conditions on the sensory analysis profile of Iberian dry-cured ham.

Table 5 shows the acceptability scores for dry-cured hams under different processing conditions given by the consumer panel. Despite the different processing conditions, the consumer panel scored the three types of ham very similarly, finding significant differences only for color, with overripeness significantly affecting these parameters. Therefore, consumer acceptability of sensory traits was similar for all the types of cured hams studied. Similar results were described by Cilla et al. [22]. Therefore, we can say that neither overripening nor the increase in temperature in the final processing phase affected the organoleptic characteristics of the salt-reduced boneless cured Iberian ham. Prolonged ripening or overripening could increase lipid oxidation, the Maillard reaction, or Strecker amino acid degradation, leading to the development of compounds responsible for the flavor of the ham [49].

## 4. Conclusions

Increasing processing time and temperature significantly increased proteolysis, but this did not result in undesirable changes in texture. No significant differences were observed in any of the analyzed parameters defining instrumental texture. The consumer panel did not detect worse texture in these hams; the texture was not significantly affected.

Furthermore, these changes in processing conditions increased the content of free amino acids, benefiting the consumer, as well as being responsible for aromas and attributes that contribute to the development of positive effects in cured ham.

These changes did not modify the sensory attributes evaluated by the participants, as no significant differences were found between the different batches—only for the instrumental color, which scored significantly lower in the overripe ham without temperature increase.

In general, despite the reduction in salt, these new processing conditions benefited the hams, with all of them obtaining very good scores in the sensory panel, with texture not being affected by increased proteolysis and with a higher content of free amino acids. In addition, increased proteolysis and NPN will lead to a higher production of peptides with possible bioactivity.

## Figures and Tables

**Table 1:** Effect of different processing conditions on the physicochemical parameters of Iberian ham. Results are expressed as means values ± SEM.

	Processing Conditions			*p*-Value	
	I	II	III	Overmaturation	Temperature
Moisture	35.39 ± 1.16 ^a^	29.81 ± 0.57 ^b^	32.13 ± 1.63 ^ab^	0.00	0.21
%NaCl	3 ± 0.16 ^a^	3.1 ± 0.18 ^a^	3.3 ± 0.18 ^a^	0.69	0.45
Fat	40.95 ± 1.64 ^a^	48.66 ± 0.11 ^b^	47.6 ± 0.68 ^b^	0.00	0.15
Ash	0.88 ± 0.01 ^a^	0.84 ± 0.01 ^b^	0.84 ± 0.01 ^b^	0.01	0.88
Protein	26.42 ± 0.22 ^a^	25.66 ± 0.33 ^b^	25.51 ± 0.11 ^b^	0.07	0.67

I: Traditional processing up to 38% weight loss at 30 °C. II: Traditional processing up to 42% weight loss at 30 °C. III: Traditional processing up to 42% weight loss with temperature increase up to 36 °C in the storage phase. SEM: standard error of mean. The *p*-value processing conditions: one-way ANOVA^a,b^ between conditions I, II, and III. Values within a row with different superscripts differ significantly at *p* < 0.05.

**Table 2:** Effect of processing conditions on free amino acid content (FAA) of Iberian dry-cured ham. Results are expressed in g/kg of dry matter as mean values ± SEM.

FAA	Processing Conditions			*p*-Value	
	I	II	III	Overmaturation	Temperature
Asp	-	-	-	-	-
Glu	2.57 ± 0.29 ^a^	3.99 ± 0.04 ^b^	3.95 ± 0.33 ^b^	0.00	0.89
Ser	1.04 ± 0.18 ^a^	0.98 ± 0.07 ^a^	1 ± 0.01 ^a^	0.86	0.85
His	0.81 ± 0.07 ^a^	1.12 ± 0.01 ^b^	1.25 ± 0.03 ^b^	0.03	0.01
Gly	2.4 ± 0.18 ^a^	3.17 ± 0.02 ^b^	3.51 ± 0.04 ^b^	0.03	0.00
Thr	0.33 ± 0.02 ^b^	0.39 ± 0.03 ^b^	1.32 ± 0.15 ^a^	0.25	0.00
Arg	0.64 ± 0.16 ^a^	0.17 ± 0.02 ^ab^	0.13 ± 0.00 ^b^	0.13	0.07
Ala	6.65 ± 0.56 ^a^	8.54 ± 0.11 ^b^	9.92 ± 0.11 ^b^	0.00	0.00
Tyr	0.47 ± 0.05 ^b^	0.7 ± 0.05 ^a^	0.49 ± 0.02 ^ab^	0.05	0.04
Cys	-	-	-	-	-
Val	2.15 ± 0.16 ^a^	2.85 ± 0.02 ^b^	3.13 ± 0.07 ^b^	0.03	0.04
Met	0.69 ± 0.06 ^a^	0.63 ± 0.02 ^a^	0.63 ± 0.08 ^a^	0.55	0.97
Trp	0.23 ± 0.02 ^b^	0.31 ± 0.01 ^a^	0.3 ± 0.01 ^ab^	0.05	0.72
Phe	1.24 ± 0.09 ^b^	1.5 ± 0.01 ^ab^	1.93 ± 0.18 ^a^	0.15	0.13
Ile	1.6 ± 0.12 ^a^	1.94 ± 0.02 ^a^	2.11 ± 0.04 ^b^	0.14	0.04
Leu	2.51 ± 0.18 ^b^	2.94 ± 0.02 ^ab^	3.39 ± 0.105 ^a^	0.23	0.02
Lys	3.18 ± 0.35 ^a^	4.05 ± 0.32 ^a^	4.39 ± 0.06 ^a^	0.25	0.50
Pro	1.17 ± 0.11 ^b^	1.51 ± 0.00 ^ab^	1.62 ± 0.01 ^a^	0.00	0.12
Total FAA	26.67	34.35	39.03	0.00	0.00

I: Traditional processing up to 38% weight loss at 30 °C. II: Traditional processing up to 42% weight loss at 30 °C. III: Traditional processing up to 42% weight loss with temperature increase up to 36 °C in the storage phase. SEM: standard error of mean. The *p*-value processing conditions: one-way ANOVA^a,b^ between conditions I, II, and III. Values within a row with different superscripts differ significantly at *p* < 0.05.

**Table 3:** Effect of processing conditions on instrumental color. Results are expressed as means values ± SEM.

	Processing Conditions			*p*-Value	
	I	II	III	Overmaturation	Temperature
L	49.2 ± 1.03 ^b^	46.03 ± 0.44 ^a^	47.54 ± 1.44 ^b^	0.01	0.33
a	28.62 ± 0.99 ^a^	30.22 ± 0.25 ^a^	28.82 ± 0.77 ^a^	0.14	0.11
b	34.75 ± 0.63 ^a^	34.47 ± 0.57 ^a^	31.93 ± 1.42 ^a^	0.74	0.12
C	45.06 ± 1.01 ^a^	45.85 ± 0.52 ^a^	43.15 ± 0.98 ^b^	0.5	0.03
h	50.59 ± 0.74 ^b^	48.73 ± 0.44 ^a^	47.75 ± 1.7 ^a^	0.05	0.59

I: Traditional processing up to 38% weight loss at 30 °C. II: Traditional processing up to 42% weight loss at 30 °C. III: Traditional processing up to 42% weight loss with temperature increase up to 36 °C in the storage phase. SEM: standard error of mean. The *p*-value processing conditions: one-way ANOVA^a,b^ between conditions I, II, and III. Values within a row with different superscripts differ significantly at *p* < 0.05.

**Table 4:** Effect of different processing conditions on instrumental texture of Iberian dry-cured ham. Results are expressed as means values ± SEM.

	Processing Conditions			*p*-Value	
	I	II	III	Overmaturation	Temperature
C1 hardness (N)	15.31 ± 2.05 ^a^	17.87 ± 2.39 ^a^	16.9 ± 1.45 ^a^	0.43	0.73
C1 hardness deformation (mm)	2.97 ± 0.00 ^a^	2.98 ± 0.00 ^a^	2.98 ± 0.00 ^a^	0.64	0.57
C1 adhesiveness (mJ)	0.03 ± 0.02 ^b^	0.24 ± 0.09 ^a^	0.36 ± 0.10 ^a^	0.04	0.38
C2 hardness (N)	14.63 ± 1.95 ^a^	16.66 ± 2.23 ^a^	15.67 ± 1.33 ^a^	0.50	0.71
C2	0.62 ± 0.04 ^a^	0.67 ± 0.03 ^a^	0.63 ± 0.03 ^a^	0.39	0.41
C2 recoverable deformation (mm)	1.43 ± 0.13 ^a^	1.50 ± 0.11 ^a^	1.45 ± 0.14 ^a^	0.65	0.78
C2 springiness (mm)	2.1 ± 0.15 ^a^	2.31 ± 0.13 ^a^	2.19 ± 0.13 ^a^	0.31	0.53
C2 gumminess (N)	9.84 ± 1.77 ^a^	12.31 ± 2.01 ^a^	10.94 ± 1.39 ^a^	0.37	0.58
C2 chewiness (mJ)	22.2 ± 5.15 ^a^	29.82 ± 5.65 ^a^	25.39 ± 4.31 ^a^	0.34	0.54

I: Traditional processing up to 38% weight loss at 30 °C. II: Traditional processing up to 42% weight loss at 30 °C. III: Traditional processing up to 42% weight loss with temperature increase up to 36 °C in the storage phase. SEM: standard error of mean. The *p*-value processing conditions: one-way ANOVA^a,b^ between conditions I, II, and III. Values within a row with different superscripts differ significantly at *p* < 0.05.

**Table 5:** Effect of different processing conditions on the sensory analysis profile of Iberian dry-cured ham. Results are expressed as means values ± SEM.

	Processing Conditions			*p*-Value	
	I	II	III	Overmaturation	Temperature
Appearance	4.15 ± 0.07 ^a^	4 ± 0.07 ^a^	4.14 ± 0.07 ^a^	0.11	0.14
Color	4.28 ± 0.06 ^b^	4.1 ± 0.07 ^a^	4.23 ± 0.07^a^	0.04	0.15
Odor	3.87 ± 0.07 ^a^	3.94 ± 0.07 ^a^	3.86 ± 0.08^a^	0.53	0.47
Texture	3.95 ± 0.07 ^a^	3.87 ± 0.07 ^a^	3.9 ± 0.08 ^a^	0.48	0.83
Salty taste	3.82 ± 0.08 ^a^	3.78 ± 0.08 ^a^	3.66 ± 0.08 ^a^	0.68	0.28
Global flavor	4.01 ± 0.07 ^a^	3.94 ± 0.07 ^a^	3.91 ± 0.07 ^a^	0.50	0.78
General acceptance	4.05 ± 0.07 ^a^	3.95 ± 0.06 ^a^	3.94 ± 0.07 ^a^	0.33	0.86

I: Traditional processing up to 38% weight loss at 30 °C. II: Traditional processing up to 42% weight loss at 30 °C. III: Traditional processing up to 42% weight loss with temperature increase up to 36 °C in the storage phase. SEM: standard error of mean. The *p*-value processing conditions: one-way ANOVA^a,b^ between conditions I, II, and III. Values within a row with different superscripts differ significantly at *p* < 0.05.

## Data Availability

The original contributions presented in the study are included in the article, further inquiries can be directed to the corresponding author.

## References

[1] Coll-Brasas E., Arnau J., Gou P., Lorenzo J.M., García-Pérez J.V., Fulladosa E. (2019). Effect of High Pressure Processing Temperature on Dry-Cured Hams with Different Textural Characteristics. Meat Sci..

[2] Petričević S., Marušić Radovčić N., Lukić K., Listeš E., Medić H. (2018). Differentiation of Dry-Cured Hams from Different Processing Methods by Means of Volatile Compounds, Physico-Chemical and Sensory Analysis. Meat Sci..

[3] Ventanas Barroso J. (2012). Jamón Ibérico y Serrano: Fundamentos de la Elaboración y de la Calidad.

[4] Bermúdez R., Franco D., Carballo J., Lorenzo J.M. (2014). Physicochemical Changes during Manufacture and Final Sensory Characteristics of Dry-Cured Celta Ham. Effect of Muscle Type. Food Control.

[5] Pérez-Santaescolástica C., Carballo J., Fulladosa E., Garcia-Perez J.V., Benedito J., Lorenzo J.M. (2018). Effect of Proteolysis Index Level on Instrumental Adhesiveness, Free Amino Acids Content and Volatile Compounds Profile of Dry-Cured Ham. Food Res. Int..

[6] Toldrá F. (2006). The Role of Muscle Enzymes in Dry-Cured Meat Products with Different Drying Conditions. Trends Food Sci. Technol..

[7] Zhang J., Zhen Z., Zhang W., Zeng T., Zhou G. (2009). Effect of Intensifying High-Temperature Ripening on Proteolysis, Lipolysis and Flavor of Jinhua Ham. J. Sci. Food Agric..

[8] Zhang J., Wang L., Liu Y., Zhu J., Zhou G.H. (2006). Changes in the Volatile Flavour Components of Jinhua Ham during the Traditional Ageing Process. Int. J. Food Sci. Technol..

[9] Benedini R., Parolari G., Toscani T., Virgili R. (2012). Sensory and Texture Properties of Italian Typical Dry-Cured Hams as Related to Maturation Time and Salt Content. Meat Sci..

[10] Buscailhon S., Monin G., Cornet M., Bousset J. (1994). Time-Related Changes in Nitrogen Fractions and Free Amino Acids of Lean Tissue of French Dry-Cured Ham. Meat Sci..

[11] Toldrá F., Flores M. (1998). The Role of Muscle Proteases and Lipases in Flavor Development during the Processing of Dry-Cured Ham. Crit. Rev. Food Sci. Nutr..

[12] Virgili R., Saccani G., Gabba L., Tanzi E., Soresi Bordini C. (2007). Changes of Free Amino Acids and Biogenic Amines during Extended Ageing of Italian Dry-Cured Ham. LWT—Food Sci. Technol..

[13] Muñoz-Rosique B., Hernández-Correas N., Abellán A., Bueno E., Gómez R., Tejada L. (2023). Influence of Pig Genetic Line and Salt Reduction on Peptide Production and Bioactivity of Dry-Cured Hams. Foods.

[14] Taormina P.J. (2010). Implications of Salt and Sodium Reduction on Microbial Food Safety. Crit. Rev. Food Sci. Nutr..

[15] Hunter R.W., Dhaun N., Bailey M.A. (2022). The Impact of Excessive Salt Intake on Human Health. Nat. Rev. Nephrol..

[16] Zhao G.M., Zhou G.H., Wang Y.L., Xu X.L., Huan Y.J., Wu J.Q. (2005). Time-Related Changes in Cathepsin B and L Activities during Processing of Jinhua Ham as a Function of pH, Salt and Temperature. Meat Sci..

[17] Harkouss R., Safa H., Gatellier P., Lebert A., Mirade P.-S. (2014). Building Phenomenological Models That Relate Proteolysis in Pork Muscles to Temperature, Water and Salt Content. Food Chem..

[18] Harkouss R., Chevarin C., Daudin J.-D., Sicard J., Mirade P.-S. (2018). Development of a Multi-Physical Finite Element-Based Model That Predicts Water and Salt Transfers, Proteolysis and Water Activity during the Salting and Post-Salting Stages of the Dry-Cured Ham Process. J. Food Eng..

[19] Armenteros M., Aristoy M.-C., Barat J.M., Toldrá F. (2012). Biochemical and Sensory Changes in Dry-Cured Ham Salted with Partial Replacements of NaCl by Other Chloride Salts. Meat Sci..

[20] Toldrá F., Aristoy M.-C., Flores M. (2000). Contribution of Muscle Aminopeptidase to Flavor Development in Dry Cured Ham. Food Res. Int..

[21] Benedini R., Raja V., Parolari G. (2008). Zinc-Protoporphyrin IX Promoting Activity in Pork Muscle. LWT—Food Sci. Technol..

[22] Cilla I., Martínez L., Beltrán J.A., Roncalés P. (2005). Factors Affecting Acceptability of Dry-Cured Ham throughout Extended Maturation under “Bodega” Conditions. Meat Sci..

[23] Ruiz J., López R.C., Rojas M.T.A., Tejeda J.L., Córdoba J.J., Martín L.M. (1998). Influencia de las condiciones de elaboración sobre la proteolisis durante la maduración del jamón ibérico. Food Sci. Technol. Int. = Cienc. Y Tecnol. De Aliment. Int..

[24] Soresi Bordini C. (2004). Effect of Ageing Time on the Analytical and Sensory Parameters of Parma Ham [Emilia-Romagna]. Ind. Conserve (Italy).

[25] Mora L., Fraser P., Toldrá F. (2013). Proteolysis Follow-up in Dry-Cured Meat Products through Proteomic Approaches. Food Res. Int..

[26] Zhou G.H., Zhao G.M. (2007). Biochemical Changes during Processing of Traditional Jinhua Ham. Meat Sci..

[27] Meat and Meat Products—Determination of Moisture Content (Reference Method).

[28] Official Methods of Analysis, 22nd Edition (2023). https://www.aoac.org/official-methods-of-analysis/.

[29] Abellán A., Salazar E., Vázquez J., Cayuela J.M., Tejada L. (2018). Changes in Proteolysis during the Dry-Cured Processing of Refrigerated and Frozen Loin. LWT.

[30] Harkouss R., Mirade P.-S., Gatellier P. (2012). Development of a Rapid, Specific and Efficient Procedure for the Determination of Proteolytic Activity in Dry-Cured Ham: Definition of a New Proteolysis Index. Meat Sci..

[31] Meat and Meat Products—Determination of Chloride Content—Part 1: Volhard Method.

[32] Folch J., Lees M., Stanley G.H.S. (1957). A Simple Method for the Isolation and Purification of Total Lipides from Animal Tissues. J. Biol. Chem..

[33] Ash of Meat—$14.15: AOAC Official Method.

[34] Abellán A., Cayuela J.M., Pino A., Martínez-Cachá A., Salazar E., Tejada L. (2012). Free Amino Acid Content of Goat’s Milk Cheese Made with Animal Rennet and Plant Coagulant. J. Sci. Food Agric..

[35] Muñoz-Rosique B., Salazar E., Tapiador J., Peinado B., Tejada L. (2022). Effect of Salt Reduction on the Quality of Boneless Dry-Cured Ham from Iberian and White Commercially Crossed Pigs. Foods.

[36] Trinh T. (2012). On the Texture Profile Analysis Test. https://ibf.iuh.edu.vn/wp-content/uploads/2019/09/tpatest.pdf.

[37] Análisis Sensorial. Metodología. Guía General.

[38] Análisis Sensorial. Directrices Para La Util…..

[39] Alía A., Rodríguez A., Andrade M.J., Gómez F.M., Córdoba J.J. (2019). Combined Effect of Temperature, Water Activity and Salt Content on the Growth and Gene Expression of Listeria Monocytogenes in a Dry-Cured Ham Model System. Meat Sci..

[40] Coutron-Gambotti C., Gandemer G. (1999). Lipolysis and Oxidation in Subcutaneous Adipose Tissue during Dry-Cured Ham Processing. Food Chem..

[41] Vestergaard C.S., Schivazappa C., Virgili R. (2000). Lipolysis in Dry-Cured Ham Maturation. Meat Sci..

[42] Andres A.I., Ventanas S., Ventanas J., Cava R., Ruiz J. (2005). Physicochemical Changes throughout the Ripening of Dry Cured Hams with Different Salt Content and Processing Conditions. Eur. Food Res. Technol..

[43] Costa-Corredor A., Serra X., Arnau J., Gou P. (2009). Reduction of NaCl Content in Restructured Dry-Cured Hams: Post-Resting Temperature and Drying Level Effects on Physicochemical and Sensory Parameters. Meat Sci..

[44] Martín L., Córdoba J.J., Antequera T., Timón M.L., Ventanas J. (1998). Effects of Salt and Temperature on Proteolysis during Ripening of Iberian Ham. Meat Sci..

[45] Rodríguez-Nuñez E., Aristoy M.-C., Toldrá F. (1995). Peptide Generation in the Processing of Dry-Cured Ham. Food Chem..

[46] Zhou C.-Y., Wu J.-Q., Tang C.-B., Li G., Dai C., Bai Y., Li C.-B., Xu X.-L., Zhou G.-H., Cao J.-X. (2019). Comparing the Proteomic Profile of Proteins and the Sensory Characteristics in Jinhua Ham with Different Processing Procedures. Food Control.

[47] Guo X., Wang Y., Lu S., Wang J., Fu H., Gu B., Lyu B., Wang Q. (2021). Changes in Proteolysis, Protein Oxidation, Flavor, Color and Texture of Dry-Cured Mutton Ham during Storage. LWT.

[48] Cittadini A., Domínguez R., Gómez B., Pateiro M., Pérez-Santaescolástica C., López-Fernández O., Sarriés M.V., Lorenzo J.M. (2020). Effect of NaCl Replacement by Other Chloride Salts on Physicochemical Parameters, Proteolysis and Lipolysis of Dry-Cured Foal “Cecina”. J. Food Sci. Technol..

[49] Flores M., Grimm C.C., Toldrá F., Spanier A.M. (1997). Correlations of Sensory and Volatile Compounds of Spanish “Serrano” Dry-Cured Ham as a Function of Two Processing Times. J. Agric. Food Chem..

[50] Sanabria C., Martín-Alvarez P., Carrascosa A. (2004). Colour and Moisture Changes during the Manufacture of Iberian Dry-Cured Ham Caused by Some Biotic and Abiotic Factors. Food Sci. Technol. Int.—Food Sci. Technol. Int..

[51] Ruiz J., Serra X., Gou P., Arnau J. (2006). Effect of Proteolysis Index on Texture of Dry-Cured Ham. Arch. Latinoam. Prod. Anim..

